# Comparison of antioxidant, phenolic profile, melatonin, and volatile compounds of some selected plant samples

**DOI:** 10.1002/fsn3.4334

**Published:** 2024-07-10

**Authors:** Halil İbrahim Binici, İhsan Güngör Şat, Bilal Yilmaz

**Affiliations:** ^1^ Department of Nutrition and Dietetics, Faculty of Health Sciences İstanbul Esenyurt University İstanbul Türkiye; ^2^ Department of Food Engineering, Faculty of Agriculture Ataturk University Erzurum Türkiye; ^3^ Department of Analytical Chemistry, Faculty of Pharmacy Ataturk University Erzurum Türkiye

**Keywords:** antioxidant, GC–MS, melatonin, phenolic profile

## Abstract

It was aimed to examine the antioxidant, phenolic profile, and volatile compound contents of seven different aromatic plant samples (broccoli, yarpuz, walnut leaves, marshmallow, wild clary, harmala, and common yarrow) collected from Adilcevaz district of Bitlis province in Türkiye. Gas chromatography–mass spectrometry (GC–MS) device was used for the volatile profile of the plant varieties. The most abundant volatile compounds were generally heptacosane compound that showed anticancer and antimicrobial effects. Piperitone oxide was detected only in yarpuz samples. Phenolic content and antioxidant activity of the plant samples were found to be highly significant (*p* < .01). When the amounts of total flavonoids were ranked from the largest to the smallest according to plant varieties, it was determined as wild clary > walnut leaves > yarpuz > common yarrow > marshmallow > harmala > broccoli. According to both antioxidant activity methods, wild clary had the lowest IC_50_ (half‐maximal inhibitory concentration) value, i.e. the highest antioxidant activity. The highest amount of epicatechin was determined in yarpuz (898.30 μg/g) and the lowest amount of epicatechin was determined in wild clary (86.09 μg/g). No epicatechin was detected in the other four plant samples. Among the samples, melatonin hormone was detected only in common yarrow, harmala, and broccoli and the highest value was determined in common yarrow (3996.27 ng/g). Therefore, it shows that plant samples are rich sources of phytochemicals that can play an important role in preventing the progression of many diseases related to oxidative stress in traditional medicine treatment as functional food sources.

## Graphical abstract

Plant samples are rich sources of phytochemicals that can play an important role in preventing the progression of many oxidative stress‐related diseases in traditional medicine treatment.

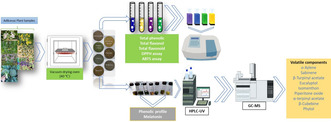

## 1 INTRODUCTION

Fruits and vegetables are known as a natural source of antioxidants that protect against free radicals and prevent many chronic diseases, such as cardiovascular, cancer, diabetes, and Alzheimer's disease. Fruits and vegetables also contain carotenoids, vitamins, flavonoids, phenolic compounds, dietary glutathione, and endogenous metabolites (Zhang et al., 2004). In general, wild plants are said to be a natural source of antioxidants for human health. The antioxidant properties of plants are due to their phytochemical contents, such as phenolic acids, flavonoids, carotenoids, and tocopherols (Çömlekcİoğlu & Kutlu, 2018). Therefore, fruit and vegetable consumption is of great importance for human health in the prevention of many diseases (Syed et al., 2023). Broccoli (*Brassica oleracea* L. var. Italica) is a perennial plant with stalked leaves belonging to the Brassicaceae family. Broccoli is consumed as a fresh vegetable and is known as an important source of dietary fiber components containing flavone glycosides as well as vitamin carotenoids. The pharmaceutical importance of broccoli vegetable is increasing and it is known that this pharmaceutical effect consists of antioxidant, anticancer, anti‐amnesic, antimicrobial, and antidiabetic properties. It is reported that the daily broccoli consumption will support the immune system, bone health, eye diseases, and digestive system (Syed et al., 2023; Zhang et al., 2004). Yarpuz (*Mentha pulegium* L.) is a medicinal and aromatic plant species rich in secondary metabolites (Ayaz & Memon, 2021; Çöteli et al., 2013). This plant, which is widely used in traditional and alternative medicine, is reported to have anti‐inflammatory, antiemetic, carminative, diaphoretic, analgesic, antispasmodic, antitussive, and stimulant effects (Gülçin et al., 2020). At the same time, *Mentha* plant has pharmaceutical effects and is widely used as a flavoring agent for raw and processed foods, such as salads, soups, herbal teas, cheese, and bread (Fontefrancesco et al., 2019). Walnut (*Juglans regia* L.) is a species belonging to the Juglandaceae family known for its high nutritional value and is rich in bioactive components (Salık & Çakmakçı, 2023; Yilmaz et al., 2023). The walnut leaf is also used in traditional medicine due to its blood sugar lowering effect, aromatic profile, antidiabetic, antidiarrheal, and antiparasitic properties (Delaviz et al., 2017). Marshmallow (*Althaea officinalis* L.) plant in the Malvaceae family is an annual or perennial plant (Karimi et al., 2022). It is reported that marshmallow plant has been used in traditional medicine for thousands of years and this plant is used in the treatment of wounds and bruises. It is known that marshmallow is widely used for upper respiratory tract infections, sore throat, cough, abdominal pain, and eczema (Özyazgan et al., 2023). Wild clary (*Salvia verbenaca* L.) in the Lamiaceae family is known as a plant that prevents coronary heart disease, hypertension, hepatocirrhosis, chronic kidney disease, and inflammatory disorders for human health (Vezza et al., 2023). Wild clary has high biological activity and functional properties (Özdek & Fakir, 2019). The plant harmala (*Peganum harmala* L.) in the Zygophyllaceae family is a perennial hairless erect plant. It is reported that the plant is widely used in traditional medicine and is effective in cardiovascular diseases, nervous system disorders, antimicrobial and immune system (Moloudizargari et al., 2013; Yuca et al., 2022). Common yarrow (*Achillea millefolium* L.), the best known and most common species of the Asteraceae family, is a therapeutic perennial herbaceous plant with pharmaceutical value. Common yarrow has been used as a traditional herbal medicine for more than 3000 years. This plant rich in bioactive components is known to have antibacterial, antifungal, antiviral, insecticidal, and antioxidant properties (Far et al., 2023).

The aim of this study is to have information about the analyses of total phenolic content (TPC), antioxidant capacity, phenolic profile, and volatile compounds of broccoli, yarpuz, walnut leaves, marshmallow, wild clary, harmala, and common yarrow, which are folk plants grown in Türkiye and used for healing purposes in various forms against diseases. Since there is no study with such a variety of analyses of these plants used for therapeutic purposes, it is thought that our study will contribute to the literature.

## 2 MATERIALS AND METHODS

### 2.1 Collecting plant species

In June 2023, the plants were collected from the vicinity of Mount Süphan at an altitude of 2150 m in Adilcevaz district of Bitlis. Species identification of the collected plant samples was made according to Davis' flora of Türkiye (Davis et al., 1988). About 500 ± 0.1 g of all plant samples was collected, dried in a vacuum oven at 40°C, and stored (2014 ND, Arçelik) at −18°C until analysis.

### 2.2 Determination of total phenolic content

One gram of the plant samples was weighed, 20 mL of 80:20% methanol:water was added and centrifuged in an ultrasonic water bath (Bandelin Sonorex Super RK 100 H, Germany) for 15 min. After centrifugation (Hettich Zentrifugen Mikro 22R‐1110, Germany) at 4200 *g* for 15 min, the filtrate was filtered through Whatman No: 40 and this filtrate was used. The total phenolic content was determined according to Şat et al. (2023), 0.100 mL of these plant samples was taken and 0.800 mL 0.2 N Folin–Ciocalteu reagent (FCR), 0.800 mL sodium carbonate (Na_2_CO_3_) (10%), and 0.800 mL distilled water were added, respectively, and the final volume was completed to 2.5 mL. The amounts of total phenolic content were measured in a spectrophotometer (PG Instrument TV‐60) at 760 nm by drawing the gallic acid (GA) standard and kept in the dark for 30 min.

### 2.3 Determination of total flavonol content

One gram of the plant samples was weighed, 20 mL of 80:20% methanol: water was added and centrifuged in an ultrasonic water bath (Bandelin Sonorex Super RK 100 H, Germany) for 15 min. After centrifugation (Hettich Zentrifugen Mikro 22R‐1110, Germany) at 4200 *g* for 15 min, the filtrate was filtered through Whatman No: 40 and this filtrate was used. Total flavonol (Tflavonol) content was determined according to Binici et al. (2021). The total flavonol content was given as quercetin equivalent (QE) and measured in a spectrophotometer (PG Instrument TV‐60) at 415‐nm wavelength.

### 2.4 Determination of total flavonoid content

One gram of the plant samples was weighed, 20 mL of 80:20% methanol: water was added and centrifuged in an ultrasonic water bath (Bandelin Sonorex Super RK 100 H, Germany) for 15 min. After centrifugation (Hettich Zentrifugen Mikro 22R‐1110, Germany) at 4200 *g* for 15 min, the filtrate was filtered through Whatman No: 40 and this filtrate was used. Total flavonoid content was determined according to Binici et al. (2021). Total flavonoid content (Tflavonoid) amount was given as quercetin equivalent (QE) and measured in a spectrophotometer (PG Instrument TV‐60) at 510‐nm wavelength.

### 2.5 Determination of DPPH assay

One gram of the plant samples was weighed, 20 mL of 80:20% methanol: water was added and centrifuged in an ultrasonic water bath (Bandelin Sonorex Super RK 100 H, Germany) for 15 min. After centrifugation (Hettich Zentrifugen Mikro 22R‐1110, Germany) at 4200 *g* for 15 min, the filtrate was filtered through Whatman No: 40 and this filtrate was used. 2,2‐Diphenyl‐1‐picrylhydrazyl (DPPH) radical scavenging activity was performed according to Binici et al. (2021). The DPPH radical scavenging activity values were given as IC_50_ values and measured in a spectrophotometer (PG Instrument TV‐60) at 517‐nm wavelength.

### 2.6 ABTS assay

One gram of the plant samples was weighed, 20 mL of 80:20% methanol:water was added and centrifuged in an ultrasonic water bath (Bandelin Sonorex Super RK 100 H, Germany) for 15 min. After centrifugation (Hettich Zentrifugen Mikro 22R‐1110, Germany) at 4200 *g* for 15 min, the filtrate was filtered through Whatman No: 40 and this filtrate was used. 2,2‐Azino‐bis‐3‐ethylbenzothiazoline‐6‐sulfonic acid (ABTS) scavenging ability of plant samples was determined according to Zor et al. (2022). Briefly, a 7 mM ABTS solution was prepared by adding potassium persulfate (K_2_S_2_O_8_) (2.45 mM) and stirred overnight in the dark. Absorbance was then measured at 734 nm to give a wavelength of 0.700 ± 0.025 nm. Samples were prepared at concentrations of 10–30 μg/mL, 0.500 mL of ABTS radical was added, and the final volume was made up to 2 mL with distilled water.

### 2.7 Determination of melatonin by HPLC

One gram of sample was kept in 10 mL of acetonitrile (ACN)/water (45/55) solvent in an ultrasonic water bath (Bandelin Sonorex Super RK 100 H, Germany) for 1 h. It was centrifuged (Hettich Zentrifugen Mikro 22R‐1110, Germany) at 2400 × *g* for 10 min. The supernatant was passed through a 0.45‐μm filter. One milliliter of this supernatant was taken. The solvent was evaporated under N_2_ (nitrogen) gas. The remaining extract was dissolved in 0.1 mL of ACN and applied to high‐performance liquid chromatography–ultraviolet (HPLC–UV) device. Areas were found from the chromatogram. They were replaced on the calibration curve and given as nanograms per gram (ng/g). This chromatographic system was equipped with a quaternary pump (G7111A), auto injector (G7129A), and ultraviolet (UV) detector (G71144A). Reversed‐phase C18 column (5 μm, 250 × 4.6 mm), mobile phase was ACN:water (45:55) with a flow rate of 1 mL/min, and the injection volume was 20 μL. At 220‐nm wavelength, retention times and ultraviolet (UV) spectra were compared and confirmed.

### 2.8 Determination of phenolic profile by HPLC

Phenolic profiles of plant samples were determined according to Alwazeer et al. (2023).

### 2.9 Determination of volatile components by GC–MS

Before injection, 1 g of each dried plant sample was dissolved in 10 mL of ethyl acetate and centrifuged (Hettich Zentrifugen Mikro 22R‐1110, Germany) at 4200 × *g* for 15 min. The supernatant was filtered through a 0.22‐μm filter (Millipore, Carrigtwohill, Ireland) and 10 mL of the filtered mixture was transferred into amber‐colored bottles. Gas chromatography–mass spectrometry (GC–MS) investigations were completed on an Agilent 7820A Gas chromatograph (GC) system equipped with 7673 series autosampler and Agilent ChemStation and 5977 series mass selective detector (MSD). HP‐5 MS segment with 0.25‐μm film thickness (30 m × 0.25 mm I.D., Agilent Technologies, USA) was utilized for separation. The temperatures of the inlet and transfer line were 250 and 300°C, respectively. Chemical compounds were identified using the National Institute of Standards and Technology (NIST) GC–MS library and reference standard materials.

### 2.10 Color analysis

The color analysis of the plants was determined according to Lu et al. (2007). The color was measured by Minolta colorimeter (ChromaMeter, CR‐200) in triplicate.

### 2.11 Statistical analysis

Statistical analyses were calculated using SPSS software (IBM SPSS 25). Values are presented as arithmetic mean ± standard deviation (±SD). The statistical significance of the differences was evaluated by one‐way analysis of variance (ANOVA) test.

## 3 RESULTS AND DISCUSSION

In this study, samples of selected different plants were studied. TPC, Tflavonol, Tflavonoid, antioxidant (ABTS IC_50_, DPPH IC_50_) activity, and color values of the samples are shown in Table 1. The TPC values of the samples ranged between 19.94 and 65.25 mg GAE/g (milligrams of gallic acid equivalent per gram), and the highest total phenolic content (65.25 mg GAE/g) was found in wild clary plant. Total flavonol and total flavonoid contents of the wild clary plant were 4.97 and 211.71 mg QE/g (milligrams of quercetin per gram), respectively. When the flavonoid amounts were ranked from the largest to the smallest according to plant varieties, it was determined as wild clary > walnut leaves > yarpuz > common yarrow > marshmallow > harmala > broccoli. Farhat et al. (2013), in a study conducted to determine the phenolic and antioxidant properties of different types of *Salvia* species, determined the total phenolic content of *Salvia argentea* and *Salvia officinalis* species as 67.67–72.02 mg GAE/g and 112.93–161.37 mg GAE/g, respectively. Plants are rich in bioactive compounds and functionally contain phytochemical components with pharmacological effects. Some of the secondary metabolites of plants show natural antioxidant properties and these secondary metabolites are reported to reduce the risk and progression of many diseases, such as cancer, cardiovascular diseases, and neurodegenerative diseases. In addition, flavonoid substances in plants are considered as promising compounds among secondary metabolites of plants (Al‐Owaisi et al., 2014). Due to the limited data on these varieties in the literature, it shows that it is good in terms of specificity. The reason why wild clary plant is higher than other plant varieties is thought to be due to the species, variety, and growing conditions of the plant varieties.

**TABLE 1:** Total phenolic content (TPC), total flavonol, total flavonoid, and antioxidant activities (DPPH IC_50_ (μg/mL) and ABTS IC_50_ (μg/mL)) of plant samples.

Analyses/samples	Broccoli	Yarpuz	Walnut leaves	Marshmallow	Wild clary	Harmala	Common yarrow	Sig.
TPC (mg GAE/g)	19.94 ± 0.21^f^	31.52 ± 2.25^c^	26.24 ± 0.90^d^	41.38 ± 2.74^b^	65.25 ± 0.53^a^	22.76 ± 0.24^e^	34.21 ± 1.89^c^	*
Tflavonol (mg QE/g)	1.09 ± 0.01^g^	4.11 ± 0.01^c^	3.70 ± 0.00^d^	3.20 ± 0.32^e^	4.97 ± 0.01^a^	2.03 ± 0.00^f^	4.58 ± 0.00^b^	*
Tflavonoid (mg QE/g)	7.31 ± 0.25^g^	83.95 ± 1.05^c^	100.85 ± 0.19^b^	49.43 ± 0.62^e^	211.71 ± 0.98^a^	14.17 ± 0.33^f^	80.70 ± 0.99^d^	*
DPPH IC_50_ (μg/mL)	5328.29 ± 409.44^a^	486.45 ± 0.72^c^	373.79 ± 0.73^cd^	446.73 ± 1.75^cd^	160.42 ± 0.21^d^	1206.87 ± 21.30^b^	445.45 ± 0.34^cd^	*
ABTS IC_50_ (μg/mL)	1099.16 ± 34.86^a^	167.98 ± 4.60^d^	226.25 ± 0.46^c^	93.48 ± 5.61^e^	45.81 ± 0.02^f^	436.02 ± 3.39^b^	190.32 ± 1.73^d^	*

*Note*: a–g: Means with different letters are statistically different (*p* < .05).

* *p* < .01.

The IC_50_ values of DPPH radical scavenging activity of the samples were found to be between 160.42 and 5328.29 μg/mL and the IC_50_ values of ABTS radical scavenging activity were found to be between 45.81 and 1099.16 μg/mL. There is an inverse relationship between IC_50_ values and antioxidant activity. In other words, the antioxidant activities of the samples with low IC_50_ values are high. Accordingly, as per both antioxidant activity methods, wild clary has the lowest IC_50_ value, i.e. the highest antioxidant activity (Table 1). Among the plant samples, broccoli had the lowest antioxidant activity of 1099.16 μg/mL (Table 1). With a long‐term history of use in traditional medicine, plants represent a major source for the discovery and investigation of new drugs by pharmaceutical sciences (Al‐Nemari et al., 2020). The aim of the present study was to investigate the biological activities of extracts from various plants and to examine their potential use as complementary medicine or new remedies for the treatment of diseases, especially cancer.

Melatonin hormone, also known as N‐acetyl‐5‐methoxytryptamine, is a neurotransmitter that first appeared in 1958. Melatonin hormone is a hormone effective on human biorhythms (Şat & Binici, 2021). Melatonin content of plant samples varied between nd‐3996.27 ng/g. Among the samples, melatonin hormone was detected only in common yarrow, harmala, and broccoli, and the highest value was determined in common yarrow (3996.27 ng/g) (Table 2).

**TABLE 2:** Melatonin and phenolic profiles of plant samples characterized by HPLC.

Compounds/samples	Broccoli	Yarpuz	Walnut leaves	Marshmallow	Wild clary	Harmala	Common yarrow	Sig.
Melatonin (ng/g)	406.16 ± 4.09^c^	nd	nd	nd	nd	3531.16 ± 35.31^b^	3996.27 ± 39.96^a^	**
Gallic acid (μg/g)	26.23 ± 0.14^d^	28.28 ± 0.11^d^	26.87 ± 0.04^d^	99.46 ± 0.35^b^	1218.98 ± 24.55^a^	nd	71.64 ± 0.12^c^	**
Chlorogenic acid (μg/g)	20.74 ± 0.08^c^	23.79 ± 0.13^b^	nd	nd	131.34 ± 0.27^a^	nd	nd	**
Catechin (μg/g)	nd	nd	nd	nd	470.71 ± 0.14^a^	74.10 ± 0.12^b^	nd	**
Epicatechin (μg/g)	nd	898.30 ± 0.19^a^	nd	nd	86.09 ± 0.13^c^	nd	658.31 ± 0.10^b^	**
Rutin (μg/g)	nd	nd	nd	36.74 ± 0.13^a^	nd	nd	32.33 ± 0.13^b^	**
p‐Coumaric acid (μg/g)	nd	nd	nd	25.55 ± 0.19^b^	54.46 ± 0.02^a^	nd	23.60 ± 0.08^c^	**
Trans‐Ferulic acid (μg/g)	nd	115.51 ± 0.19^a^	nd	nd	33.08 ± 0.17^b^	nd	nd	**
Rosmarinic acid (μg/g)	72.75 ± 0.13^c^	nd	912.39 ± 2.37^a^	76.38 ± 0.12^b^	24.59 ± 0.15^e^	nd	38.92 ± 0.11^d^	**
Quercetin (μg/g)	nd	nd	35.51 ± 0.17^b^	nd	38.91 ± 0.12^a^	nd	nd	**

*Note*: a–d: Means with different letters are statistically different (*p* < .05).

Abbreviation: nd, Not determined.

** *p* < .01.

When the phenolic profile amounts of the plant samples were analyzed, the highest amount of gallic acid was found in wild clary (1218.98 μg/g). No gallic acid was detected in harmala plant. The amount of chlorogenic acid was determined as 131.34 μg/g in wild clary (Table 2). Chlorogenic acid was detected in only three plant samples (wild clary, yarpuz, and broccoli). Catechin was detected in only two samples (wild clary and harmala). The highest catechin content was detected in wild clary. The highest amount of epicatechin was determined in yarpuz (898.30 μg/g) and the lowest amount of epicatechin was determined in wild clary (86.09 μg/g). No epicatechin was detected in the other four plant samples. In two samples, rutin was detected (marshmallow and common yarrow). All compounds, except rutin (gallic acid, chlorogenic acid, catechin, epicatechin, p‐coumaric acid, trans‐Ferulic acid, rosmarinic acid, and quercetin), were detected in the wild clary sample. Only catechin was detected in harmala plant. Wild clary is very rich in phenolic contents and shows antioxidant activity. In studies, various bioactive compounds obtained from plants have shown antioxidant and radical scavenging activities. It has also been reported that there is a relationship between phenolic compounds and antioxidants (Al‐Nemari et al., 2020).

The data on volatile compounds of the samples are given in Table 3. Ethyl propanoate (4.65%), ethyl acetate (0.08%), butyl acetate (9.33%), o‐xylene (0.64%), heptacosane (45.73%), 3‐ethyl‐5(2‐ethylbutyl) octadecane (0. 59%), 3‐(octadecyloxy) propyl (9E)‐octadecenoate (0.66%), (9Z)‐1‐(2‐(9X)‐9‐octadecenyl)ethoxy)‐9‐octadecene (1.07%), 15‐nonacosanone (25.65%), 14‐octacosanol (8.54%), and triacontanal (2.54%) were detected in broccoli. o‐Xylene (0.89%), sabinene (0.79%), β‐turpinyl acetate (1.48%), eucalyptol (2.32%), isomenthone (1.82%), piperitone oxide (34.11%), α‐terpinyl acetate (0. 75%), 1‐ethyldodecyl trifluoroacetate (0.80%), caryophyllene (4.69%), β‐cubebine (3.47%), phytol (1.22%), heptacosane (27.70%), and 3‐ethyl‐5(2‐ethylbutyl) octadecane (1.41%) were detected in yarpuz plant. Heptacosane (36.69%), 3‐(octadecyloxy) propyl (9E)‐octadecenoate (1.80%), 14‐octadecenal (4.12%), and alpha‐tocopherol (3.30%) were determined in walnut leaves. Heptacosane (15.46%), heneicosane (22.22%), squalene (6.95%), and octacosane (17.36%) were detected in marshmallow plant. Ethyl propanoate (19.17%), ethyl acetate (0.36%), butyl acetate (38.10%), o‐xylene (2.67%), and heptacosane (39.70%) were determined in wild clary. Ethyl propanoate (18.85%), ethyl acetate (0.82%), butyl acetate (37.56%), o‐xylene (2.52%), and heptacosane (40.24%) were detected in harmala plant. 4‐Carene (1.25%), camphene (1.38%), eucalyptol (3.04%), 2‐bomanone (10.24%), 2‐methyl‐1‐hexadecanol (1.32%), and ethyl‐5‐(2‐ethylbutyl) octadecane (2.64%) were observed in common yarrow plant. Piperitone is a compound mainly responsible for the aroma of plants. It is also known to have insecticidal, antipesticide, anti‐anorexic, and antimicrobial properties. It is also known that heptacosane has anticancer effect in a study (Khan et al., 2022).

**TABLE 3:** Volatile compounds of plant samples characterized by GC–MS.

Compounds/samples	Broccoli	Yarpuz	Walnut leaves	Marshmallow	Wild clary	Harmala	Common yarrow
Ethyl propanoate (%)	4.65	6.07	16.69	11.09	19.17	18.85	11.52
Ethyl acetate (%)	0.08	0.07	0.33	0.20	0.36	0.82	nd
Butyl acetate (%)	9.33	12.43	34.67	25.21	38.10	37.56	23.49
o‐Xylene (%)	0.64	0.89	2.40	1.52	2.67	2.52	1.60
Sabinene (%)	nd	0.79	nd	nd	nd	nd	nd
β‐Turpinyl acetate (%)	nd	1.48	nd	nd	nd	nd	nd
Eucalyptol (%)	nd	2.32	nd	nd	nd	nd	nd
Isomenthone (%)	nd	1.82	nd	nd	nd	nd	nd
Piperitone oxide (%)	nd	34.11	nd	nd	nd	nd	nd
α‐Terpinyl acetate (%)	nd	0.75	nd	nd	nd	nd	nd
1‐Ethyldodecyl trifluoroacetate (%)	nd	0.80	nd	nd	nd	nd	nd
Caryophyllene (%)	nd	4.69	nd	nd	nd	nd	nd
β‐Cubebine (%)	nd	3.47	nd	nd	nd	nd	nd
Phytol (%)	nd	1.22	nd	nd	nd	nd	nd
Heptacosane (%)	45.73	27.70	36.69	15.46	39.70	40.24	43.53
3‐Ethyl‐5(2‐ethylbutyl) octadecane (%)	0.59	1.41	nd	nd	nd	nd	nd
3‐(Octadecyloxy) propyl (9E)‐octadecenoate (%)	0.66	nd	1.80	nd	nd	nd	nd
14‐Octadecenal (%)	nd	nd	4.12	nd	nd	nd	nd
Alpha‐tokopherol (%)	nd	nd	3.30	nd	nd	nd	nd
Heneicosane (%)	nd	nd	nd	22.22	nd	nd	nd
Squalene (%)	nd	nd	nd	6.95	nd	nd	nd
Octacosane (%)	nd	nd	nd	17.36	nd	nd	nd
4‐Carene (%)	nd	nd	nd	nd	nd	nd	1.25
Camphene (%)	nd	nd	nd	nd	nd	nd	1.38
Eucalyptol (%)	nd	nd	nd	nd	nd	nd	3.04
2‐Bomanone (%)	nd	nd	nd	nd	nd	nd	10.24
2‐Methyl‐1‐hexadecanol (%)	nd	nd	nd	nd	nd	nd	1.32
Ethyl‐5‐(2‐ethylbutyl) octadecane (%)	nd	nd	nd	nd	nd	nd	2.64
(9Z)‐1‐(2‐(9X)‐9‐octadecenyl)ethoxy)‐9‐Octadecene (%)	1.07	nd	nd	nd	nd	nd	nd
15‐Nonacosanone (%)	25.65	nd	nd	nd	nd	nd	nd
14‐Octacosanol (%)	8.54	nd	nd	nd	nd	nd	nd
Triacontanal (%)	2.54	nd	nd	nd	nd	nd	nd

Abbreviation: nd, Not determined.

## 4 CONCLUSION

The current study compared the total phenolic content, antioxidant capacity, phenolic profile, and volatile compound levels of seven different plants belonging to various families. Regarding the analyzed properties, wild clary generally showed higher values compared to other studied plants, proposing it as a promising functional plant. Melatonin was detected only in harmala, common yarrow, and broccoli with lower levels in the latter compared to other plants. Additionally, different levels of volatile components have been observed in the studied plants. According to the obtained results, it could be concluded that the studied plant species could be beneficial against oxidative stress‐related diseases as well as could be suggested as promising functional foods.

## AUTHOR CONTRIBUTIONS

Conceptualization, İ.G.Ş.; methodology, İ.G.Ş., H.İ.B., and B.Y.; validation, İ.G.Ş.; investigation, H.İ.B. and B.Y.; writing original draft preparation, H.İ.B. and B.Y.; writing review and editing, İ.G.Ş.; supervision, İ.G.Ş. All authors have read and agreed to the published version of the manuscript.

## ACKNOWLEDGEMENTS

Not available.

## FUNDING INFORMATION

No funding was received from any foundation.

## CONFLICT OF INTEREST STATEMENT

The authors declare no conflicts of interest.

## Data Availability

The data that support the findings of this study are available on request from the corresponding author. The data are not publicly available due to privacy or ethical restrictions.
